# Endoscopic Prevalence of Barrett’s Esophagus in a Predominantly African American and Hispanic Population

**DOI:** 10.7759/cureus.113784

**Published:** 2026-08-01

**Authors:** Kesiena Akpoigbe, Joan Culpepper-Morgan, Angela Barnes, Ifeoma Kwentoh, Kenneth J Vega

**Affiliations:** 1 Internal Medicine, NYC Health + Hospitals/Harlem, New York, USA; 2 Gastroenterology, NYC Health + Hospitals/Harlem, New York, USA; 3 Gastroenterology and Hepatology, Prisma Health Midlands, Columbia, USA

**Keywords:** barrett’s esophagus, disparity, ethnicity, incidence, race, survival

## Abstract

Background and aim

Barrett’s esophagus (BE) is a well-known premalignant disorder. Investigators have shown that the prevalence of histologically confirmed BE varies among ethnic groups in the US. However, these studies were not performed at institutions that predominantly serve US minority populations. The aim of this study was to assess the prevalence of histologically confirmed BE at an institution that serves a predominantly African American (AA) and Hispanic American patient population.

Methods

The Harlem Hospital endoscopy and pathology databases were searched to identify all patients who underwent esophagogastroduodenoscopy (EGD) with biopsy and had histologically confirmed BE. Histological confirmation of BE was determined by the presence of intestinal metaplasia and Alcian blue-stained goblet cells in biopsies obtained from salmon-colored esophageal mucosa. Demographic data collected included age, sex, race/ethnicity, BMI, history of gastroesophageal reflux disease, endoscopic BE length, presence of hiatal hernia, esophagitis, or esophageal ulcer, presence or absence of dysplasia, proton pump inhibitor use, active *Helicobacter pylori* infection, and smoking and alcohol use.

Results

A total of 3,012 patients underwent EGD during the study period. Esophageal biopsy was performed on salmon-colored esophageal mucosa suspicious for BE in 18 individuals. BE was histologically confirmed in eight patients: five AAs, two Hispanics, and one non-Hispanic White (nHW). The overall prevalence of BE was 0.2%, with a lower prevalence observed among AA patients (0.3%) and Hispanic patients (0.1%) than among nHW patients (3.3%, p < 0.05). Four patients had dysplasia (three with low-grade dysplasia and one with high-grade dysplasia; three AA and one Hispanic).

Conclusions

The prevalence of BE in our predominantly minority population was lower than that observed among nHW patients, consistent with previous literature. Investigations at the microbiome and genetic levels are needed to explain the observed disparity in BE prevalence among ethnic groups.

## Introduction

Barrett’s esophagus (BE) is a premalignant lesion of the esophagus with an estimated prevalence of 5.6% in the US [1]. It is associated with esophageal carcinoma, with an annual risk of progression of 0.25-0.5% in patients without dysplasia and 6% in those with dysplasia [2-4]. BE increases the risk of developing esophageal adenocarcinoma (EAC) by at least sevenfold and possibly as much as 30- to 40-fold [3,4]. The incidence of BE has increased over the past four decades [5].

Several risk factors have been associated with BE. Obesity, particularly central obesity, increases the risk of BE [1,6,7]. A linear relationship has been established between BE and central obesity, with a significantly greater dysplastic component observed at three- to five-year follow-up [5,8]. Smoking has also been associated with BE, with a prevalence of up to 12% among smokers compared with nonsmokers [2,9,10]. The combination of alcohol use and smoking has also been reported to contribute to the development of BE [11]. Furthermore, sex differences have been noted in the development of BE, with males more likely to develop BE than females [10,12-14].

In addition, differences in the endoscopic prevalence of BE have been observed among racial/ethnic groups [15]. BE is more common in the non-Hispanic White (nHW) population [15-20]. African American (AA) ethnicity has not been associated with the development of BE [16]. Abrams et al. estimated the prevalence of BE in AAs and Hispanics to be low, with the odds of developing BE reported as 0.34 for AA and 0.38 for Hispanics compared with nHWs [15]. However, nearly 30% of subjects in that investigation were classified as either “other” or “unknown” race, introducing the potential for misclassification bias. Of note, these studies comparing racial prevalence were conducted in predominantly nHW communities. To date, there are no data on the endoscopic prevalence of BE at an institution serving a predominantly AA/Hispanic community. The aim of this study was to assess the prevalence of BE at a facility serving a predominantly AA/Hispanic population.

This article was previously presented as a meeting abstract at the 2023 ACG Annual Scientific Meeting on October 22, 2023.

## Materials and methods

Study design and patients

This was a retrospective cross-sectional study of patients at Harlem Hospital. Harlem Hospital is a community hospital that serves a diverse minority population in New York City. This population includes a greater proportion of AA (65%) and Hispanic (30%) patients. All patients who underwent esophagogastroduodenoscopy (EGD) with biopsy between January 2018 and January 2022, for any indication, were included to determine the endoscopic prevalence of BE in our predominantly AA and Hispanic population. A total of 3,012 patients underwent EGD during this period. Esophageal biopsy was performed in 888 patients. Random biopsies for histological examination were obtained during the procedures in patients suspected of having BE or salmon-colored mucosa. De-identified data for all patients were obtained from the electronic medical record system.

Demographic variables were recorded for each patient, including age at diagnosis, sex, race, and ethnicity. The histological diagnosis of BE was extracted from the pathology reports. Other histological information collected included the presence of esophagitis, dysplasia, and esophageal carcinoma. Endoscopic findings, including the length of the BE segment, were also recorded. Long-segment BE (LSBE) was defined as a BE length greater than 3 cm. Other information extracted included risk factors for BE, such as BMI, *Helicobacter pylori *infection, smoking, and alcohol use.

Statistical analysis

Statistical analysis was performed using de-identified data extracted from the electronic medical record. The data were sorted, coded, and matched with the corresponding demographic characteristics and risk factor information. The statistical analysis was based on the type of variable. Continuous variables, such as age and BMI, were summarized using frequency, mean, and SD across the different racial/ethnic groups. Categorical variables were summarized using frequencies and percentages. The chi-square test and one-way analysis of variance were used to assess associations among race/ethnicity, risk factors, and BE. A two-sided p-value < 0.05 was considered statistically significant. Statistical analyses were performed using Stata statistical software (StataCorp LLC, College Station, TX, USA).

## Results

A total of 3,012 patients underwent EGD for any indication during the study period. Of these, 1,497 (49.7%) were AA, 1,461 (48.5%) were Hispanic, 30 (1.0%) were nHW, and 24 (0.8%) were Asian/Native American. Esophageal biopsy was performed on 18 patients with salmon-colored mucosa suspicious for BE. Histologically confirmed BE was identified in eight patients: three AA males, two AA females, two Hispanic females, and one nHW male. The overall prevalence of BE was 0.26% (8/3,012). When BE prevalence was examined by race/ethnicity, a significantly lower prevalence was observed among AA patients (0.3% (5/1,497)) and Hispanic patients (0.1% (2/1,461)) than among nHW patients (3.3% (1/30), p < 0.05). Four patients had dysplasia. Low-grade dysplasia was identified in one AA male, one AA female, and one Hispanic female. The only patient with high-grade dysplasia that progressed to EAC was an AA male. No difference in the endoscopic prevalence of BE was observed between male (4/989 (0.4%)) and female (4/2,023 (0.2%)) patients in this study cohort (p = 0.301, Table 1).

**Table 1:** Prevalence of BE ^⁋^ Compared with White patients ^†^ Compared with male patients BE, Barrett’s esophagus

Characteristic	Number	%	p-value
Overall	8/3,012	0.2	-
Race			
White	1/30	3.3	-
Black	5/1,496	0.3	0.009^⁋^
Hispanic	2/1,462	0.1	0.000^⁋^
Asian	0/16	0	-
Native American	0/8	0	-
Sex			
Male	4/989	0.4	-
Female	4/2,023	0.2	0.301^†^

Five patients (62.5%) with BE had LSBE, with the majority being AA (3/5, 60%) compared with Hispanic patients (2/5, 40%). The remaining three patients (37.5%) had short-segment BE. Most patients with BE (6/8, 75%) had a hiatal hernia. Almost all patients with BE had concomitant esophagitis, with the exception of one AA patient (Table 2).

**Table 2:** Demographics, endoscopic findings, and risk factors of patients with BE Statistical significance was defined as p < 0.05. AA, African American; BE, Barrett’s esophagus; LSBE, long-segment Barrett’s esophagus

Ethnicity	Overall	White	AA	Hispanic	p-value
Demographics and endoscopic findings					
Number of patients	8	1	5	2	-
Age, mean ± SD	62.6 ± 10.3	73	64.2 ± 3.5	53.5 ± 20.5	0.294
Male sex, (%)	50.0	100.0	60.0	0.0	0.202
Patients with LSBE, n (%)	5 (62.5)	0 (0.0)	3 (60.0)	2 (40.0)	0.054
Patients with hiatal hernia, n (%)	6 (75.0)	0 (0.0)	4 (66.7)	2 (33.3)	0.155
Patients with esophagitis, n (%)	7 (87.5)	1 (100.0)	4 (80.0)	2 (100.0)	0.710
Patients with any dysplasia, n (%)	4 (50.0)	0 (0.0)	3 (75.0)	1 (25.0)	0.549
Patients with high-grade dysplasia/carcinoma, n (%)	1 (12.5)	0 (0.0)	1 (20.0)	0 (0.0)	0.438
Risk factors					
BMI, mean ± SD	28.9 ± 5.4	28.2	27.2 ± 4.4	33.9 ± 7.9	0.410
Proton pump inhibitor use, n (%)	7 (87.5)	0 (0.0)	5 (100.0)	2 (100.0)	0.018
*Helicobacter pylori*infection, n (%)	1 (12.5)	0 (0.0)	0 (0.0)	1 (50.0)	0.180
Smoking, n (%)	3 (37.5)	0 (0.0)	3 (60.0)	0 (0.0)	0.237
Alcohol use disorder, n (%)	0 (0.0)	0 (0.0)	0 (0.0)	0 (0.0)	-

Hispanic patients with confirmed BE had a higher mean BMI (33.9 ± 7.9) than AA patients (27.2 ± 4.4) and the single nHW patient (28.2). Most patients with BE were taking a proton pump inhibitor at the time of endoscopy. Only one patient (a Hispanic female) had *H. pylori *infection identified by EGD with biopsy. Smoking was reported by three of the eight patients with BE, all of whom were AA. None of the patients with BE had a history of alcohol use disorder (Table 2).

## Discussion

The prevalence of BE in the overall study population was 0.26%, which was much lower than the average estimated prevalence in the US [1]. The prevalence was significantly lower among AAs and Hispanics than among nHWs in our study population, consistent with previously published studies. Abrams et al. reported an endoscopic BE prevalence of 1.6% in AAs and 1.7% in Hispanics, compared with 6.1% in nHWs [15]. However, as previously stated, 28.3% of their patients may have been misclassified. Multiple investigators have reported that nondysplastic BE was more prevalent in AAs than in nHWs, whereas the current study suggests the opposite [21,22]. However, this difference did not reach statistical significance because of the small number of patients with BE.

AA ethnicity has not been established as a risk factor for the development of BE after an episode of erosive esophagitis [16]. Among veterans, the prevalence of BE was also lower in AAs than in Whites [23]. The disparity in BE prevalence currently observed in the US may be related to race or to environmental factors within the AA community compared with the nHW population [24]. These findings further support the lower prevalence observed in our study, particularly because our study was conducted at a facility that predominantly serves AAs and Hispanics in an AA/Hispanic community.

Half of the patients with BE in this study had dysplasia, including one patient with high-grade dysplasia and three with low-grade dysplasia. The prevalence of dysplasia among patients with BE was 75% in AAs and 50% in Hispanics. This contrasts with the findings of Khoury et al., who reported that dysplasia was less frequent among AAs than among nHWs [21]. The high rate of low-grade dysplasia may be related to the presence of esophagitis in all but one patient with BE, as low-grade dysplasia may be confounded by concurrent esophagitis [25,26]. Most patients with BE had LSBE and a hiatal hernia, both of which are significant risk factors for BE and dysplasia [5,12,15]. Approximately one-third of the patients with BE were active smokers. Unfortunately, the degree of smoking exposure could not be evaluated in the present investigation. Smoking has been associated with an increased risk of BE [27,28]. Although obesity is a significant risk factor for BE, the mean BMI of this study cohort was in the overweight range. Only the Hispanic patients with BE met the definition of obesity.

One explanation for the differences in prevalence may lie in the esophageal microbiome. The esophageal microbiome is greatly influenced by the oral microbiome [29]. Patterns of bacterial enrichment and depletion have been described that characterize esophageal diseases, including gastroesophageal reflux disease (GERD), BE, EAC, and squamous cell carcinoma [30]. For example, the oral microbiome of patients with BE is characterized by gram-negative bacteria, including *Fusobacterium*, *Prevotella*, and members of the Bacteroidales, as well as Firmicutes, among others [29]. The esophageal microbiome of patients with BE is also characterized by an increase in gram-negative bacteria, including *Fusobacterium*, *Neisseria*, *Campylobacter*, *Bacteroides*, Proteobacteria, and *Veillonella *taxa [31]. There is ongoing debate as to whether these changes precede the associated diseases or are a consequence of them. There is also growing evidence that significant differences in the microbiome exist among racial and ethnic groups [32]. The relative contributions of genetics and environmental factors to these differences remain to be determined. Nevertheless, it is reasonable to hypothesize that different racial and ethnic groups may have varying predispositions to developing BE and EAC versus squamous cell carcinoma because of differences in their baseline esophageal microbiome. This microenvironment, combined with environmental risk factors such as smoking, a high-fat diet, and GERD, may alter microbial populations and, in turn, influence disease expression.

The strengths of this study include its performance at a facility serving a large AA and Hispanic population, allowing for an accurate assessment of BE prevalence in these groups. As a community hospital, most patients are from the surrounding neighborhood and therefore share similar environmental exposures. This may not be the case at a tertiary referral center. Furthermore, histologic confirmation of endoscopically identified salmon-colored mucosa was required to satisfy the nationally accepted definition of BE.

There were several limitations to our study. The generalizability of the findings is limited because this was a single-center, cross-sectional study and may not reflect findings at other institutions serving predominantly AA and Hispanic populations. Second, the categorization of all Black patients as AA may not accurately represent this group. Although the AA category includes Black patients, a substantial proportion were immigrants from Africa, whereas others were Black Americans. Because Black populations are not homogeneous, this distinction was not addressed in the present study. Similarly, the Hispanic patients receiving care at our facility were predominantly Caribbean Hispanics from the Dominican Republic. Therefore, extrapolation of these findings to all Hispanic populations may not be appropriate because of the considerable genetic heterogeneity among Hispanics [15,33]. Finally, the use of BMI to estimate obesity may have underestimated visceral adiposity, as waist-to-hip ratio or waist circumference would have been more appropriate measures [34].

## Conclusions

This study further confirms that AAs and Hispanics have a lower prevalence of BE than Whites. It is possible that this difference is related to an underlying genetic predisposition, as the only White patient in the study did not have common predisposing risk factors, such as obesity, GERD, or smoking, yet still developed BE. The contributions of the broader environment, the local microenvironment, the microbiome, and epigenetic mechanisms to BE disease expression warrant further investigation. Such studies may help explain the differences in disease prevalence among AAs, Hispanics, and Whites. Although the risk of BE is lower in AAs and Hispanics than in Whites, the rate of progression from dysplastic BE to EAC in these populations remains an important area for future study.
